# Antioxidant and Antiproliferative Activities of Kale (*Brassica oleracea* L. Var. *acephala* DC.) and Wild Cabbage (*Brassica incana* Ten.) Polyphenolic Extracts

**DOI:** 10.3390/molecules28041840

**Published:** 2023-02-15

**Authors:** Dario Lučić, Iva Pavlović, Lidija Brkljačić, Sandro Bogdanović, Vladimir Farkaš, Andrea Cedilak, Lucia Nanić, Ivica Rubelj, Branka Salopek-Sondi

**Affiliations:** 1 Division of Molecular Biology, Ruđer Bošković Institute, 10000 Zagreb, Croatia; 2 Division of Organic Chemistry and Biochemistry, Ruđer Bošković Institute, 10000 Zagreb, Croatia; 3 Department of Agricultural Botany, Faculty of Agriculture, University of Zagreb, 10000 Zagreb, Croatia; 4 Centre of Excellence for Biodiversity and Molecular Plant Breeding, 10000 Zagreb, Croatia

**Keywords:** Brassicaceae, phenolic compounds, antioxidative activity, antiproliferative activity, HeLa cells

## Abstract

Brassicaceae are rich in healthy phytochemicals that have a positive impact on human health. The aim of this study was to analyze the phenolic compounds and antioxidant and anticancer potential of traditional Croatian kale (*Brassica oleracea* L. var. *acephala* DC.) and wild cabbage (*Brassica incana* Ten.) extracts. The phenolic groups and antioxidant activity were determined by spectrophotometry, selected phenolic compounds (ferulic acid, sinapic acid, salicylic acid, kaempferol, and quercetin) were analyzed by LC-MS/MS, and anticancer potential was evaluated in vitro using HeLa cells. The extracts of both plant species are rich in phenolic compounds and showed significant antioxidant activity at similar levels. LC-MS/MS detected sinapic acid as the most abundant phenolic acid, followed by ferulic acid, while salicylic acid was present at lower concentrations. A comparative analysis showed that wild cabbage contained significantly more sinapic acid, while kale contained more kaempferol and quercetin. Both *Brassica* extracts at a concentration of 50 µg mL^−1^ showed an antiproliferative effect on HeLa cells, while they did not affect the proliferation of normal human skin fibroblasts. Wild cabbage extract also showed an antiproliferative effect on HeLa cells at a lower applied concentration of 10 µg mL^−1^ of extracts. The clonogenic analysis also revealed the inhibitory effect of the extracts on HeLa colony growth.

## 1. Introduction

Cabbages (the genus *Brassica*, family Brassicaceae) include a number of species that are grown around the world and represent an important part of the human diet as fresh and preserved vegetables, vegetable oils, and condiments. All plant organs: buds, inflorescences, leaves, roots, seeds, and stems, are used as food and feed.

Kale (*B. oleracea* L. var. *acephala* DC.) has an important place in the culinary and diet of the population in Europe, Asia, and America [1]. It belongs to the Acephala group of species *B. oleracea,* which includes leafy, non-heading cabbages. It is a biennial herbaceous plant that may grow up to 2 m high in the first year of cultivation. It has extremely modest requirements for external conditions, so it is the best of all cabbages to withstand high temperatures and drought during the summer, as well as low temperatures and snow during the winter. Since it is tolerant to low temperatures, plants may serve as a fresh vegetable from late autumn to early spring the next season. In Croatia, it has been traditionally cultivated by farmers on small plots primarily for family consumption, either human or animal food [2]. Recent trends and promotion of kale as a ‘superfood’ brought kale to the menus of many restaurants, especially those focused on healthy food [1].

Kale is morphologically very similar to its ancestor, wild cabbage (*B. incana* Ten.). Wild cabbage (*B. incana*) is native to southeastern Europe, including Albania, Croatia, Greece, and Italy [3,4], while in Ukraine and Crimea it is probably naturalized [3,5]. The natural habitat of *B. incana* are the vertical rocks and the calcareous rocky slopes, from sea level up to about 600–800 m of altitude within the SE Mediterranean zone [4], suggesting its modest growing requirements and great tolerance to drought, high temperatures, and salinity. It is also edible and rich in healthy phytochemicals [6].

The health benefit of a *Brassica*-based diet was recognized from ancient times. They have been used in folk medicine to prevent sunstroke, decrease fevers, soothe sore feet, and relieve croup in children. Cabbages are furthermore known for treating different gastrointestinal and digestive ailments, curing hangovers, and reducing swelling of the breast engorgement during breastfeeding [7,8]. *Brassica* plants have become an interesting research topic recently, due to their bioactive phytochemicals (mostly glucosinolates and polyphenols) linked with different health benefits such as anticancer, antioxidant, anti-inflammatory, and cardioprotective activities [9,10,11,12,13,14]. Recent epidemiological studies have provided evidence that diets rich in *Brassica* vegetables are associated with a lower risk of the cancer of the gastrointestinal system [15,16], urinary system [17,18], lungs [19], and the reproductive system [20,21,22,23]. Anticancer activity is usually linked to healthy phytochemicals, mostly glucosinolates and polyphenols present in cruciferous vegetables [24]. Glucosinolates (GLS) are sulfur-containing compounds, especially present in Brassicaceae, in which around 96 different compounds have been identified so far [24,25]. GLS-degradation products such as isothiocyanates (ITCs), thiocyanates, nitriles, ascorbigen, indoles, etc., are considered the main ones responsible for the bioactivity of cruciferous foods. Due to the wide range of GLS breakdown products that can be found in nature, the number of cancer-preventive mechanisms reported is very diverse, from the suppression of cancer repressor gene transcription, the suppression of tumor cell growth through cell cycle blocking, to the inhibition of nucleus translocation and enzyme inhibition [24]. However, additional systematic research is necessary in this field to shed light on the mechanisms of anticancer activity of glucosinolates [13]. Polyphenols are another large group of phytochemicals present in *Brassica* species [9,26]. They have been reported to possess many useful properties for human health, including anti-inflammatory, enzyme inhibition, antimicrobial, antiallergic, vascular, and cytotoxic antitumor activity, but the most important action of polyphenolics is their antioxidant activity [27].

In this work, we aim to investigate the amounts of phenolic compounds (total phenols, flavonoids, phenolic acids, flavanols) and the level of antioxidant activity of phenolic extracts of autochthonous Croatian kale (*B. oleracea* var. *acephala*) and wild cabbage (*B. incana*) grown under control conditions. Additionally, phenolic acids (sinapic acid, ferulic acid, and salicylic acid) and flavonoids (kaempferol and quercetin), which are known to be present in cabbage and to contribute to a positive effect on health, were analyzed in detail. Furthermore, the effect of kale and wild cabbage extracts on the HeLa cells’ growth rate and the formation and survival of HeLa cell colonies were investigated.

## 2. Results

### 2.1. Kale and Wild Cabbage Plants

**Figure 1:**
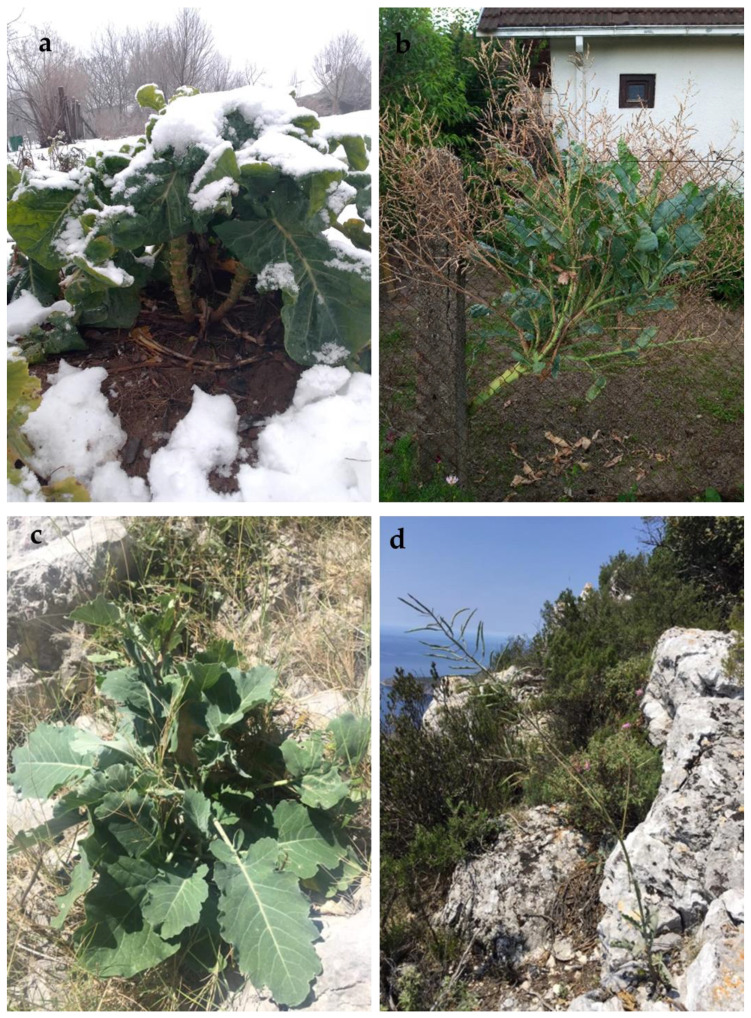
Kale (*B. oleracea* var. *acephala*) and wild cabbage (*B. incana*) grown in their natural habitat: (**a**) kale during wintertime grown on the family farm, Ogulin, Croatia (photo taken in January 2020 by Salopek-Sondi B.), (**b**) two-year-old kale bearing fruits (photo taken in June 2021 by Salopek-Sondi B.), (**c**) wild cabbage plant on the rocky slope of the Island of Vis, Croatia (photo taken in May 2020 by Bogdanović S.), and (**d**) two-year-old wild cabbage plant bearing fruits (photo taken in May 2020 by Bogdanović S.).

Grown plants of kale (*B. oleracea* var. *acephala*) and wild cabbage (*B. incana*) in their natural habitat are shown in Figure 1.

The kale stem grows to a height of 0.5–2 m in the first year of cultivation, depending on the variety. The leaves are green to gray–green, wrinkled, especially at the edges, and have very pronounced veins (Figure 1a). In the second year of cultivation, flower branches with slightly clustered inflorescences emerge from the leaf axils of the upper part of the stem, producing the fruit. Fruit are cylindrical siliques up to 12 cm long, containing two rows of seeds (Figure 1b). Wild cabbage plants are very robust with lignified stems up to 2 m high. Leaves are green, herbaceous, usually with winged petiole. The leaf lamina has rounded lobes and very visible veins (Figure 1c). The plant indumentum is dense with soft white hairs. Inflorescence with mature siliques is up to 10 cm long with a maximum 2 cm-long rostrum (Figure 1d).

**Figure 2:**
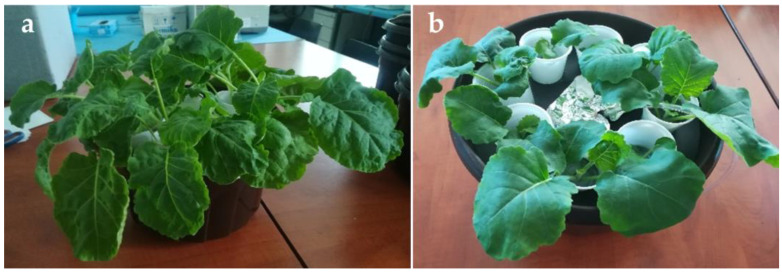
Hydroponically grown (**a**) kale (*B. oleracea* var. *acephala*) and (**b**) wild cabbage (*B. incana*).

We have grown both plant species hydroponically under controlled conditions (Figure 2). One-month-old kale plants have dark green and curled leaves on 7–10 cm-long leaf stalks (Figure 2a), while leaves of wild cabbage plants were flat, velvety due to short hairs, and on the shorter leaf stalks (3–5 cm) (Figure 2b). Leaves were harvested, freeze-dried, and used for analysis.

### 2.2. Polyphenolic Compounds

**Figure 3:**
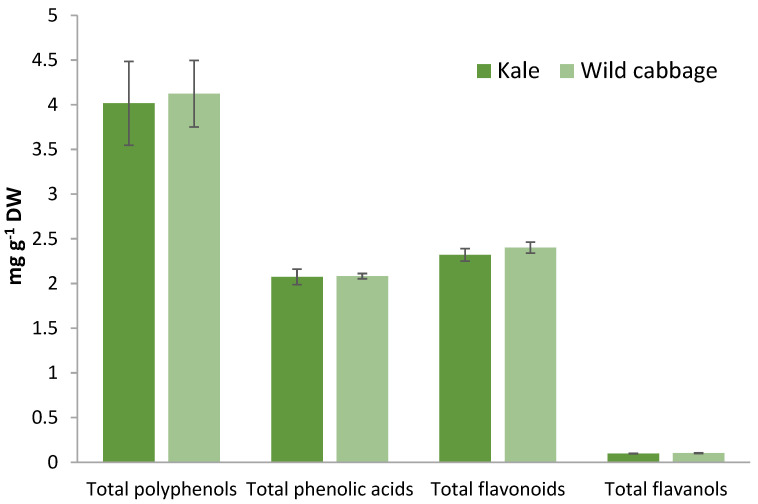
Groups of polyphenolic compounds in kale and wild cabbage (total polyphenols, total phenolic acids, total flavonoids, and total flavanols) measured by spectrophotometry. Data are mean ± SD, n = 3. Results are expressed as standard equivalent per g of dry weight (mg SE g^−1^ DW). The standards used were as follows: gallic acid (GA) for total polyphenols, caffeic acid (CA) for total phenolic acids, and catechin (C) for total flavonoids and total flavanols.

The content of polyphenolic compounds in kale and wild cabbage is shown in Figure 3. Both plants accumulated similar levels of polyphenols under our experimental conditions. Thus, the content of total polyphenols was 4.02 and 4.12 mg GAE g^−1^ DW in kale and wild cabbage, respectively. The amounts of total phenolic acids and total flavonoids were approximately the same in both *Brassica* species (2.1 mg CAE g^−1^ DW and 2.4 mg CE g^−1^ DW, respectively). Total flavanols were accumulated in lower amounts (approximately 100 µg CE g^−1^ DW in both *Brassica* plants).

Since cabbage plants are known to be rich in phenolic acids (ferulic acid (FA), sinapic acid (SiA), salicylic acid (SA)) [28] and the flavonoids kaemferol (KAE) and quercetin (QUE) [24,29], we analyzed these metabolites in detail by LC-MS/MS in kale and wild cabbage extracts. Individual metabolites were identified based on the retention time (RT) and the specific multiple reaction monitoring (MRM) transition (Table S1, Figure S1). Concentrations of selected phenolic compounds in kale and wild cabbage extracts are shown in Table 1.

As can be seen, hydroxycinnamic acids SiA and FA were highly abundant in both plant extracts. Wild cabbage contained a significantly higher level of SiA (2809.93 ± 387.56 µg g^−1^ DW) compared to kale (2211.19 ± 61.74 µg g^−1^ DW). Furthermore, both species contained a similar content of FA. Hydroxybenzoic acid, SA, was measured in a much lower amount (2.8–2.9 µg g^−1^ DW) compared to hydroxycinnamic acids. Measured flavonoids KAE and QUE were significantly higher in kale compared to wild cabbage.

### 2.3. Antioxidant Activity

**Figure 4:**
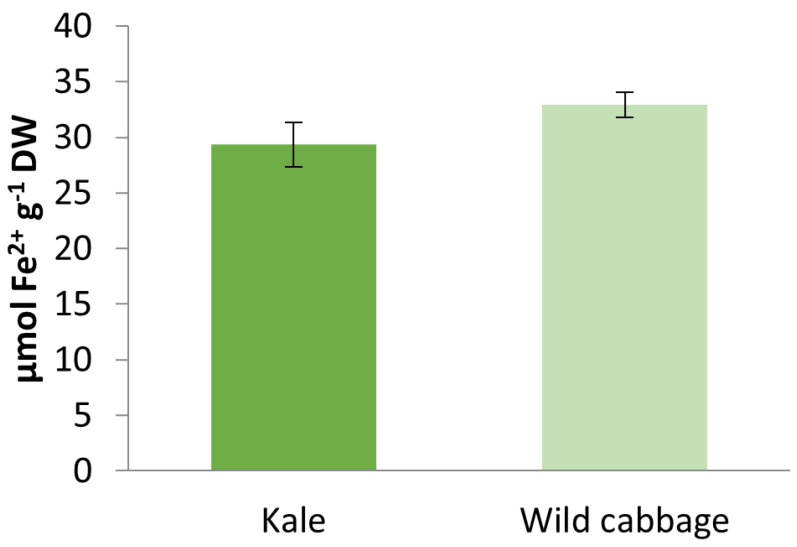
Antioxidant activity of kale (*B. oleracea* var. *acephala*) and wild cabbage (*B. incana*) methanol extracts measured by the FRAP assay. Data are mean ± SD, n = 3.

The antioxidant activity of kale and wild cabbage methanol extracts was evaluated by the FRAP method and presented in Figure 4. As can be seen, wild cabbage extract showed slightly higher, although non-significant, antioxidant activity (32.90 ± 1.11 µmol Fe^2+^ g^−1^ DW) compared to kale (29.35 ± 2.00 µmol Fe^2+^ g^−1^ DW).

### 2.4. The Effect of Kale and Wild Cabbage Plant Extracts on HeLa Cells

**Figure 5:**
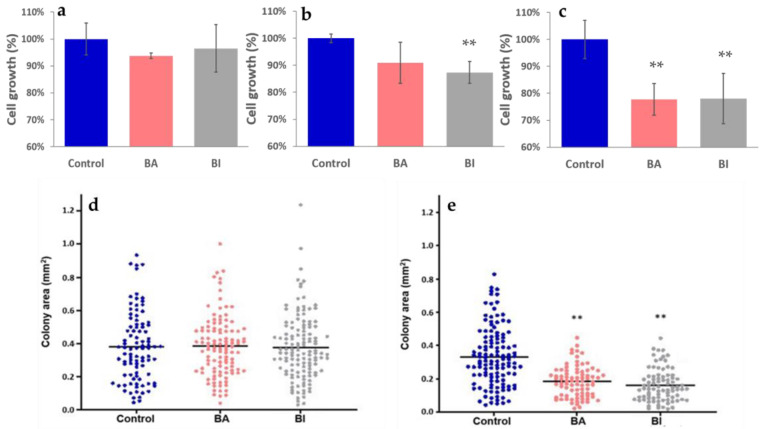
The effect of kale (*B. oleracea* var. *acephala*, BA) and wild cabbage (*B. incana*, BI) plant extracts on the growth rate of: (**a**) the normal human skin fibroblasts treated with 50 µg mL^−^^1^ of plant extracts, (**b**) HeLa cells treated with 10 µg mL^−^^1^ of plant extracts, and (**c**) HeLa cells treated with 50 µg mL^−^^1^ of plant extracts. Colony formation assay of HeLa cells was analyzed after the treatment with (**d**) 10 µg mL^−^^1^ and (**e**) 50 µg mL^−^^1^ of plant extracts. Error bars represent mean ± SD, ** *p* < 0.01.

We tested the effect of two different extract concentrations of kale and wild cabbage on the growth rate of normal human skin fibroblasts and HeLa cells, and colony-forming analysis of HeLa cells (Figure 5). Normal human skin fibroblasts treated with both concentrations of *Brassica* extracts did not show a significant difference in cell growth compared to control cells. Figure 5a shows representative results of treatments with 50 µg mL^−^^1^ extracts. When treated with 10 µg mL^−^^1^ extracts, HeLa cells exhibited a lower growth rate compared to the control. While the cells treated with kale extracts showed a slightly lower growth rate (91%), cells treated with wild cabbage extracts exhibited a significantly lower growth rate (87%) (Figure 5b). Treatment with 50 µg mL^−^^1^ extracts significantly inhibited cell growth of HeLa cells to 77% and 78% for kale and wild cabbage extracts, respectively (Figure 5c). Colony-forming analysis revealed that there was a significant decline in colony number and colony size in HeLa cells treated with 50 µg mL^−^^1^ of kale and wild cabbage extracts compared to controls (Figure 5e). The same effect was not observed in cells treated with 10 µg mL^−^^1^ extracts (Figure 5d).

## 3. Discussion

*Brassica* crops are rich in polyphenol compounds, and the content of polyphenolics depends on species/variety, plant developmental stage, cultivation, environmental conditions, etc. [7,28,30,31,32,33,34]. Kale (*B. oleracea* var. *acephala*) is considered as a “miracle food” in recent years due to its nutraceuticals value, easy cultivation, as well as high tolerance to diverse climate conditions [1,35]. In Croatia, its cultivation is still limited to small family farms. Wild cabbage (*B. incana*), although reported as edible, it is not commonly used in the human diet. It grows in its natural habitat, the vertical rocks and the calcareous rocky slopes close to the sea. Both species are hardly investigated from the metabolomics point of view, and data on metabolomics profiles are still limited as well as on the biological potential for human health [6,35]. Thus, herein, we investigated the polyphenolic contents of wild cabbage and traditional Croatian kale grown under controlled conditions, and their antioxidant and anticancer activity.

Our results showed that kale and wild cabbage contained a similar level of total polyphenols, flavonoids, phenolic acids, and flavanols. Miceli et al. [6] reported that wild cabbage (*B. incana*) contained higher levels of polyphenolic compounds compared to commonly consumed varieties of *Brassica* (such as cabbages, broccoli, cauliflower, etc.). Our previous comparative research of specialized metabolites in Chinese cabbage, white cabbage, and traditional Croatian kale emphasized kale as the richest in polyphenolic compounds [28]. As can be seen, hydroxycinnamic acids SiA and FA were highly abundant in both plant extracts, which is consistent with previously published data on kale [28] and wild cabbage [6]. Wild cabbage contained a significantly higher level of SiA compared to kale, in accordance with its antioxidant activities. Hydroxybenzoic acid, SA, was measured in a much lower amount compared to hydroxycinnamic acids, consistent with previous data [28,36]. Measured flavonoids KAE and QUE were significantly higher in kale compared to wild cabbage. While data on the bioactive compounds found in kale are available [1,35], data on the bioactive compounds of wild cabbage are very limited. The volatile compounds of wild cabbages are reported [37,38], and some publications reported the glucosinolates profile in leaves and seeds [39,40]. It was interesting that wild species showed more intense emission of volatiles, particularly isothiocyanates, than other examined Brassicaceae species [38]. Just recently, Miceli et al. [6] performed a systematic analysis of polyphenolics in wild cabbage (*B. incana*) leaves and flowers and linked that with the biological activity of wild cabbage extracts. Similar levels of polyphenolic compounds in kale and wild cabbage may be the result of their close genetic relationship and similar environmental and growth conditions.

Polyphenols are well-known as potent antioxidants capable of inhibiting the formation of ROS and quenching ROS once formed [41]. Thus, the general explanation for the beneficial properties of polyphenols is the “biochemical scavenger theory,” which claims that polyphenolic compounds neutralize or scavenge the free radicals by forming stabilized chemical complexes, thus preventing their further reactions [27]. Our results are consistent with published data that present significant antioxidant activities of kale [33,42,43] and wild cabbage extracts [6].

Plant extracts and their bioactive compounds have long been recognized for their beneficial biological properties, making them an attractive source of new therapeutic candidate compounds. Brassicaceae plants have been extensively used in traditional medicine from ancient times to the present day for different purposes (to relieve constipation, to cure hangovers and headaches, to prevent sunstroke, or to decrease fevers, etc.). In the last couple of decades, epidemiological studies have provided evidence that diets rich in cruciferous vegetables are associated with a lower risk of several types of cancer [1,7,12]. Our data demonstrated an antiproliferative effect of traditional Croatian kale and wild cabbage extracts on HeLa cells, at a higher applied concentration (50 µg mL^−^^1^ of plant extracts), while they did not affect the proliferation of normal human skin fibroblasts used as the control. Wild cabbage extract has also been shown to have an antiproliferative effect on HeLa cells at a lower concentration (10 µg mL^−^^1^ of plant extracts), maybe due to the higher level of sinapic acid and higher antioxidant activity compared to kale. Although further investigations are needed, the present study sheds light on the possible anticancer activity of kale and wild cabbage extracts. There are several reports on the cytotoxic effect of cabbages [44,45], broccoli [46,47], and curly kales [48,49,50] linked to the richness in polyphenolic compounds and antioxidant activity. There is only rare evidence of anticancer activity of wild *Brassica* species [6] and flat-leaved kale [36,47]. Although glucosinolates are mostly reported as bioactive molecules with anticancer activity [24,25], Furdak et al. [51] showed that the cytotoxic effects of a number of plant extracts, including white cabbage extracts, also correlated with their polyphenol content. Sinapic acid and ferulic acid, found at a high level in *Brassica* species, are strong antioxidants with reported anticancer activity [52,53,54,55]. Furthermore, flavonoids, particularly quercetin and kaempferol, have also been reported as powerful antioxidants and anticarcinogenic phytochemicals found in cabbages [56,57]. The synergistic effect of these polyphenols is probably responsible, in a part, for the antiproliferative effects of kale and wild cabbage extracts. Further metabolic research and screening of the potentially healthy compounds in Brassicaceae species, particularly glucosinolates and polyphenols, are necessary, and in line with Mandrich’s and Caputo’s claim that, “Brassica metabolites are emerging as new weapons for anticancer therapeutics” [58].

## 4. Materials and Methods

### 4.1. Plant Growth

Kale seeds (*B. oleracea* var. *acephala*) were obtained from the Srđan Franić family farm, Vrgorac, Croatia. Wild cabbage (*B. incana*) was identified by Sandro Bogdanović, who collected the seeds in their natural habitat on the island of Vis, Croatia. For hydroponic growth, seeds were germinated on 1% agar plates. Several-day-old seedlings were placed in a homemade hydroponic growth system supplying commercially available nutrient solutions (Flora Series and GHE Hydroponics) according to the manufacturer’s instructions, as described earlier [59]. Plants were grown in dark pots in a growing chamber at 22 °C, with 16/8 h light (115 mmol m^−^^2^ s^−^^1^)/dark cycles for 4 weeks. Leaves were then harvested in liquid nitrogen, stored at −80 °C, and then freeze-dried (Lyovac GT 2, Steris GmbH) until analysis.

### 4.2. Analysis of Polyphenolic Compounds

#### 4.2.1. Extraction of Polyphenolic Compounds

For spectrophotometry, lyophilized plant material (30 mg) was extracted in 80% methanol (1 mL) as described earlier [28]. Supernatants were evaporated in a vacuum concentrator (Thermo Scientific Savant SPD1010 Integrated SpeedVac System) at 40 °C. Pellets were subjected to hot alkaline hydrolysis, as described earlier [33]. After adjusting the pH of the samples to 2–3, ethyl acetate was added (3 × 500 μL), the organic phase containing phenolic compounds was collected, and solvents were evaporated. Dry residues were weighed and resolved in 100% methanol until the final concentration of 10 mg mL^−^^1^.

Samples for LC-MS/MS analysis of selected phenolic compounds (salicylic acid, ferulic acid, sinapic acid, kaempferol, and quercetin) were prepared the same way as described, except the extraction was performed in 80% MeOH with the addition of 20 μg of the anthracene-9-carboxylic acid (ANT) as an internal standard. Dry residues were stored at −20 °C prior to analysis. All extractions were performed in triplicates.

#### 4.2.2. Spectrophotometry of Polyphenolic Compounds and Antioxidant Activity

Methanol extracts were used for spectrophotometry analysis (BioSpec-1601 E, Shimadzu) of phenolic compounds and antioxidant activity. Total phenolics (TP) content was determined using Folin–Ciocalteu reagent [60] and results are expressed as equivalents of gallic acid per dry weight (mg GAE g^−^^1^ DW). For total flavonoids (TF) determination, we used the method with AlCl_3_ according to Zhishen et al. [61], and results are expressed as catechin equivalents per dry weight (mg CE g^−^^1^ DW). The total flavanol (TFL) content was determined using the p-dimethylaminocinnamaldehyde (DMACA) method [62], and results are expressed as catechin equivalents per dry weight (CE g^−^^1^ DW). Total phenolic acids (TPA) were determined using Arnow’s reagent according to the European Pharmacopoeia [63] and expressed as equivalents of caffeic acid (mg CAE g^−^^1^ DW). Antioxidant activity was evaluated by the ferric reducing/antioxidant power assay (FRAP) [64] and results were expressed as μmol Fe^2+^ g^−^^1^ DW.

#### 4.2.3. LC-MS/MS Analysis of Selected Polyphenolic Compounds

HPLC-grade standards sinapic acid (SiA), *trans*-ferulic acid (t-FA), quercetin (QUE), and salicylic acid (SA) were purchased from Sigma Aldrich (Saint Louis, USA), kaempferol (KAE) was from Carl Roth (Karlsruhe, Germany), and the internal standard antracene-9-carboxilic acid (ANT) was from Alfa Aesar (Haverhill, Massachusetts, USA). MiliQ^®^ water (18.2 MΩcm^−1^; purified by MiliQ water purification system (Millipore, Bedford, MA, USA)) and HPLC gradient-grade methanol (MeOH) (J.T. Baker, Center Valley, PA, USA) were used with analytical-grade formic acid (Acros Organics, Geel, Belgium) for mobile phase preparation.

LC-MS/MS analysis of selected polyphenolic compounds (SiA, FA, SA, KAE, QUE) was performed as described earlier [36]. In brief, the analyte stock solutions (1 mg mL^−^^1^) in methanol were used for the preparation of calibration curves. Samples were dissolved in 400 μL of the mobile phase and a volume of 20 µL was injected on the Zorbax XDP C18 column (75 × 4.6 mm, 3.5 μm particle size) (Agilent Technologies Inc., Palo Alto, CA, USA). LC-MS/MS analysis was carried out using an Agilent Technologies 1200 series HPLC system equipped with a binary pump, a vacuum membrane degasser, an automated autosampler, and an injector interfaced with a 6420 triple-quadrupole mass spectrometer with an electrospray ionization source (ESI) (Agilent Technologies Inc., Palo Alto, CA, USA). Solvents for the analysis were 0.1% formic acid in water (solvent A) and methanol (solvent B). The gradient was applied as follows: 0 min 60% A, 3–12 min 60% A-30% A, 5–20 min 30% A-0% A, 20–25 min 0% A, and 25.1–30 min 60% A. The flow rate was 0.3 mL/min. The electrospray ionization source was operated in negative mode and samples were detected in the multiple reaction monitoring (MRM) mode with a dwell time of 10 ms per MRM transition. The de-solvation gas temperature was 350 °C with a flow rate of 6.0 L min^−^^1^. The capillary voltage was 3.5 kV. The collision gas was nitrogen. Fragmentor voltages were: for SiA at 100 V, for KAE and QUE at 135 V, and for *trans*-FA, ANT, and SA at 70 V. MRM transitions of precursor to product ion pairs are presented in Table S1. Collision energy was set for QUE and SA at 15 V, for KAE at 20 V, for SiA and *trans*-FA at 10 V, and for ANT at 5 V. All data acquisition and processing were performed using Agilent MassHunter software.

### 4.3. Biological Activity Analysis of Brassica Plant Extracts

#### 4.3.1. Cell Culture and Treatments

The human cervical carcinoma epithelioid cell line HeLa was obtained from the American Type Culture Collection (ATCC). Normal human skin fibroblast cells were isolated from the upper arm of a 7-year-old female donor at the Neurochemical Laboratory, Department of Chemistry and Biochemistry School of Medicine, University of Zagreb [65]. Cells were grown in Dulbecco’s modified Eagle’s medium (DMEM; Sigma, St. Louis, MO, USA), supplemented with 10% fetal bovine serum (FBS; Gibco) in a humidified 37 °C incubator with 5% CO_2_. HeLa cells were cultured in 6-well plates (15,000 cells per well) in a 2 mL medium. Cells were treated with 10 mg mL^−^^1^ of kale or wild cabbage plant extracts prepared in 100% methanol so that the final concentrations per well were 10 or 50 µg mL^−^^1^ of extracts and 0.1% and 0.5% methanol in 2 mL of medium, respectively. Control wells were treated with 0.1% or 0.5% methanol in the medium. To test each extract concentration, two independent experiments were performed in duplicates. The treatments started 24 h following the cell seeding. The medium was changed, and treatments were repeated every 48 h until the fastest-growing wells reached 90% confluency. All cells were then fixed in 1% glutaraldehyde in 1 mL of medium for 10 min at room temperature. Plates were then washed three times in PBS and were ready for the following cell density quantification using crystal violet.

#### 4.3.2. Cell Growth Analysis

The crystal violet assay was used to quantitate the relative cell growth in the culture. Crystal violet stains the nucleus dark blue and the cytoplasm light blue as it binds to proteins and DNA. Following fixation, 0.2% crystal violet solution was added to HeLa cells and incubated for 10 min at room temperature. Excess dye was removed by extensive washing with deionized water and plates were air-dried. Bound dye was solubilized in 1 mL of 10% acetic acid and shaken for 20 min at room temperature. The optical density of dye extracts was measured in 500 µL at a 600 nm wavelength.

#### 4.3.3. Colony-Forming Analysis

For the colony-forming assay, HeLa cells were cultured in 6-well plates (100 cells per well) and treated with 10 mg mL^−1^ of kale or wild cabbage plant extracts prepared in 100% methanol so that the final concentrations per well were 10 or 50 µg mL^−1^ of extracts and 0.1% and 0.5% methanol in 2 mL of medium, respectively. Following the 7-day treatment, cells were stained with 0.2% crystal violet solution as described earlier. Formed colonies were documented and images were quantitated in the ImageJ program. Between 83 and 129 colonies were analyzed per treatment.

### 4.4. Statistical Analysis

Statistical analysis was performed in Excel using Student’s t-test (*p* < 0.05). Error bars represent mean ± SD. Significant differences are indicated by asterisks (* *p* < 0.05, ** *p* < 0.01, and *** *p* < 0.001).

## Figures and Tables

**Table 1:** Selected phenolic compounds measured by LC-MS/MS. Data are mean ± SD, n = 3. Asterisk (*) indicates a significant difference in polyphenolic compounds in wild cabbage compared to kale in a Student’s t-test (0.05 > *p* > 0.01).

Phenolic Compounds (µg g^−1^ DW)	Kale	Wild Cabbage
*cis, trans*-Sinapic acid (SiA)	2211.19 ± 61.74	2809.93 ± 387.56 *
*trans*-Ferulic acid (FA)	1247.97 ± 13.74	1204.123 ± 148.18
Salicylic acid (SA)	2.95 ± 0.24	2.82 ± 0.34
Kaempferol (KAE)	0.42 ± 0.10	0.24 ± 0.07 *
Quercetin (QUE)	0.20 ± 0.02	0.17 ± 0.01 *

## Data Availability

The raw data that support the findings of this study are available from the authors (B.S.-S. and I.R.) upon reasonable request.

## Supplementary Materials

The following supporting information can be downloaded at: https://www.mdpi.com/article/10.3390/molecules28041840/s1, Figure S1: Representative MRM chromatograms of standard mixture: anthracene-9-carboxylic acid (AN), ferulic acid (FE), kaempferol (KAM), quercetin (KVE), salicylic acid (SA), and sinapic acid (SIN) (upper), and representative MRM chromatograms of kale (*Brassica oleracea* var. *acephala*) (lower). Table S1: List of phenolic compounds and the internal standard used (anthracene-9-carboxylic acid) determined by UPLC-MS/MS, and the MRM transitions of precursor to product ion pairs.

## Sample Availability

Samples of the extracts are not available. Samples of *Brassica* seeds are available from the authors (B.S.-S. and S.B.) upon reasonable request.
