# Supplementary material for: Antioxidant and Antiproliferative Activities of Kale (Brassica oleracea L. Var. acephala DC.) and Wild Cabbage (Brassica incana Ten.) Polyphenolic Extracts
Source: Molecules. 2023 Feb 15;28(4):1840. doi: 10.3390/molecules28041840 (PMC9958672; doi:10.3390/molecules28041840)
Supplement: Supplementary file 1 [file molecules-28-01840-s001.zip › molecules-2206010-supplementary.pdf]

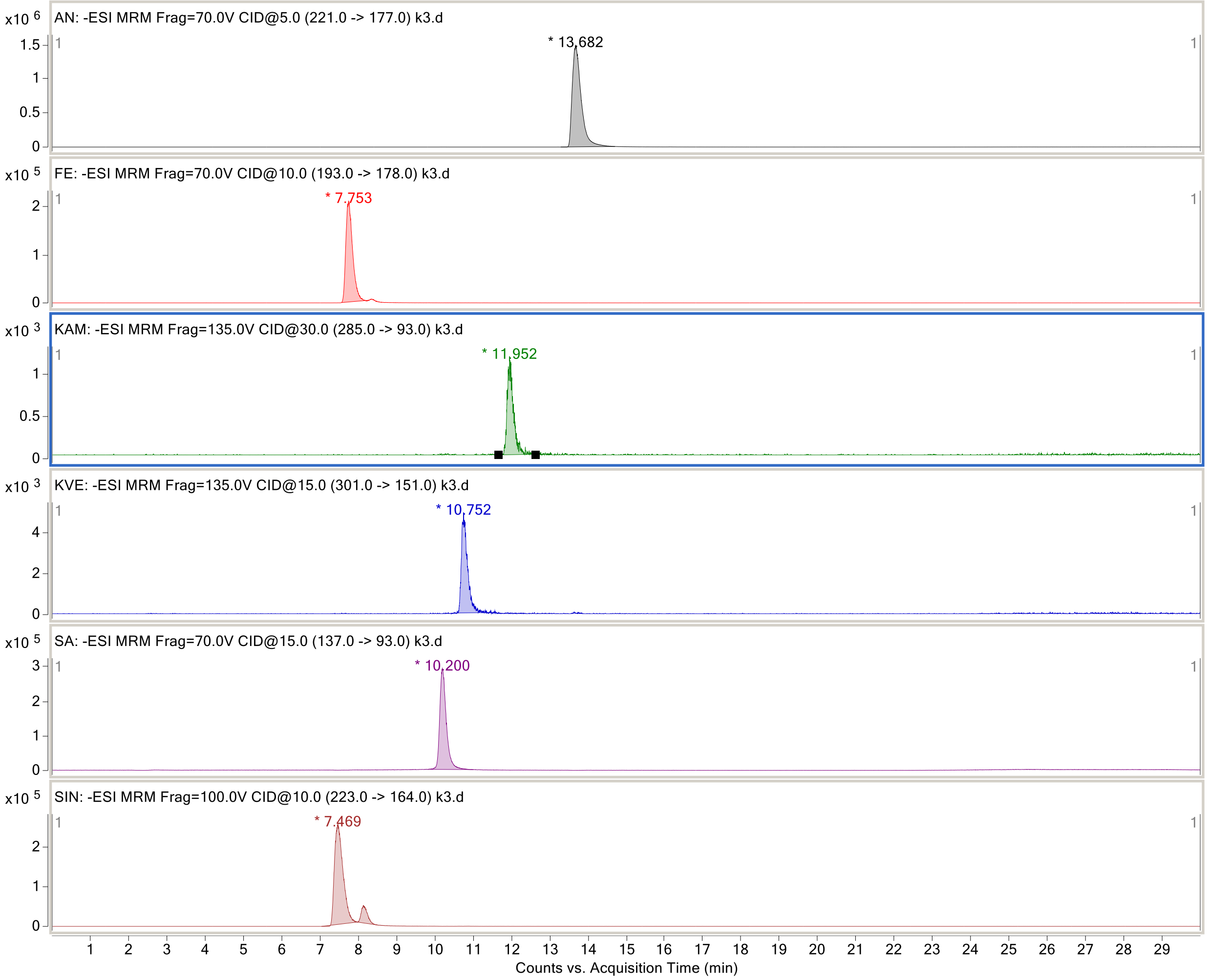

x10 6
AN: -ESI MRM Frag=70.0V CID@5.0 (221.0 -> 177.0) k3.d
* 13.682
x10 5
FE: -ESI MRM Frag=70.0V CID@10.0 (193.0 -> 178.0) k3.d
* 7.753
x10 3
KAM: -ESI MRM Frag=135.0V CID@30.0 (285.0 -> 93.0) k3.d
* 11.952
x10 3
KVE: -ESI MRM Frag=135.0V CID@15.0 (301.0 -> 151.0) k3.d
* 10.752
x10 5
SA: -ESI MRM Frag=70.0V CID@15.0 (137.0 -> 93.0) k3.d
* 10.200
x10 5
SIN: -ESI MRM Frag=100.0V CID@10.0 (223.0 -> 164.0) k3.d
* 7.469
Counts vs. Acquisition Time (min)

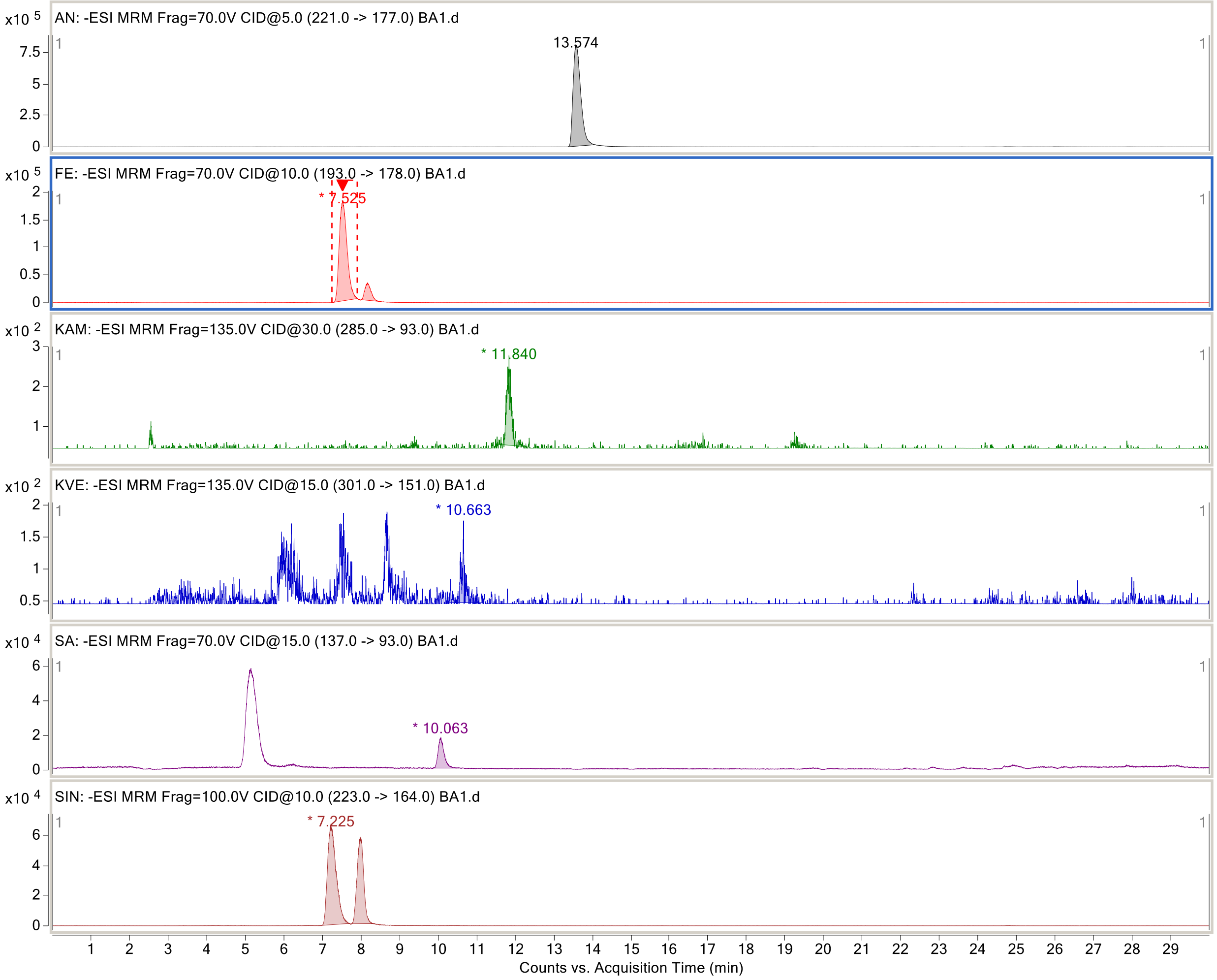


Figure S1. Representative MRM chromatograms of standard mixture: Anthracene-9-carboxylic acid (AN), Ferulic acid (FE), Kaemferol (KAM), Quercetin (KVE), Salicylic acid (SA), Sinapic acid (SIN) (upper), and representative MRM chromatograms of kale (*Brassica oleracea* var. *acephala*) (lower).

Table S1. List of phenolic compounds and used internal standard (anthracene-9-carboxylic acid) determined by UPLC–MS/MS, and the MRM transitions of precursor to product ion pairs.

| Compound (Abbreviation) | MRM (*m*/*z*) |
|---|---|
| *cis, trans*- Sinapic acid (SiA) | 223.0 -> 164.0 |
| *trans*-Ferulic acid (FA) | 193.0 -> 178.0 |
| Salicylic acid (SA) | 137.0 -> 93.0 |
| Quercetin (QUE) | 301.0 -> 151.0 |
| Kaemferol (KAE) | 285.0 -> 93.0 |
| Anthracene-9-carboxylic acid (AN) | 221.0 -> 177.0 |
